# Thyroid Function in Patients with Type 2 Diabetes Mellitus and Diabetic Nephropathy: A Single Center Study

**DOI:** 10.1155/2018/9507028

**Published:** 2018-12-02

**Authors:** Wei Zhao, Xinyu Li, Xuhan Liu, Lu Lu, Zhengnan Gao

**Affiliations:** Department of Endocrinology, Dalian Municipal Central Hospital Affiliated of Dalian Medical University, Dalian 116033, China

## Abstract

**Background:**

Diabetes mellitus is a common metabolic disease and the prevalence is increasing rapidly. Thyroid disorders including subclinical hypothyroidism (SCH) and low triiodothyronine (T3) syndrome are frequently observed in diabetic patients. We conducted a study to explore thyroid function in patients with type 2 diabetes mellitus (T2DM) and diabetic nephropathy (DN).

**Methods:**

We included 103 healthy volunteers, 100 T2DM patients without DN, and 139 with DN. Physical examinations including body mass index and blood pressure and laboratory measurements including renal function, thyroid function, and glycosylated hemoglobin were conducted.

**Results:**

Patients with DN had higher thyroid stimulating hormone (TSH) levels and lower free T3 (FT3) levels than those without DN (*p* < 0.01). The prevalence of SCH and low FT3 syndrome in patients with DN was 10.8% and 20.9%, respectively, higher than that of controls and patients without DN (*p* < 0.05). Through Pearson correlation or Spearman rank correlation analysis, in patients with DN, there were positive correlations in TSH with serum creatinine (*r* = 0.363,* p* = 0.013) and urinary albumin-to-creatinine ratio (*r* = 0.337,* p* = 0.004), and in FT3 with estimated glomerular filtration rate (eGFR) with statistical significance (*r* = 0.560,* p* < 0.001).

**Conclusions:**

High level of TSH and low level of FT3 were observed in T2DM patients with DN. Routine monitoring of thyroid function in patients with DN is necessary, and management of thyroid dysfunction may be a potential therapeutic strategy of DN.

## 1. Introduction

Diabetes mellitus (DM) is a common metabolic disease characterized by hyperglycemia and metabolic disturbances of carbohydrates, proteins, and lipids principally caused by pancreatic *β*-cell dysfunction, hyperglucagonemia, and increased renal glucose reabsorption [1, 2]. DM is rapidly becoming one of the major health problems worldwide. The estimated global prevalence of DM was 2.8% in 2000 and was predicted to increase to 4.4% in 2030 [3]. China is among the countries with the highest prevalence, with the estimated prevalence of 10.9% in adults [4]. Diabetic nephropathy (DN) is a chronic microvascular complication of diabetes and is one of the main causes of renal failure [5], which shortens lifespan and aggravates healthcare burdens. However, achieved effects on the prevalence and the prognosis of DN are not satisfactory [6]. Therefore, it is necessary to explore the underlying pathogenesis and potential management of DN.

Thyroid hormones are essential for metabolism and energy homeostasis and participate in insulin action and glucose regulation [7–10]. Previous studies reported higher prevalence rates of thyroid disorders in diabetic patients compared with nondiabetic individuals, and overt hypothyroidism was frequently observed in type 2 diabetes mellitus (T2DM) [11, 12]. Moreover, subclinical hypothyroidism (SCH), a pathological status defined as an elevated serum thyroid stimulating hormone (TSH) value with normal concentrations of free thyroid hormones [13], is receiving increasing concerns in recent years. A meta-analysis reported that the pooled prevalence of SCH in T2DM patients was 10.2% and Chinese patients with the prevalence of 18.9% were more frequently affected compared with those from other included countries [14]. Meanwhile, high levels of TSH and low levels of free triiodothyronine (FT3) within the normal range were related to a higher risk of chronic kidney disease (CKD) [15]. Also, low level of serum FT3 was found to be independently associated with urinary protein in T2DM patients [16].

In addition, genetic and environmental factors are related to the prevalence of diabetes and the effects of potential risks (such as thyroid dysfunction) on the processes of diabetic complications; although the mechanisms still remain unclear, the geographical variabilities in manifestations exist [14, 17]. In Taiwan, a cross-sectional study suggested that SCH was an independent risk (OR 3.15, 95% CI 1.48–6.69) of DN in patients with T2DM [18]. Another investigation in the south of China showed that patients with DN had lower FT3 levels than those without DN [16]. In the present study, we explored the relationship between levels of serum thyroid hormones and DN in the northeast of China, where both T2DM and thyroid disorders are common [19].

## 2. Patients and Methods

### 2.1. Patients

A total of 135 healthy volunteers aged 18-80 years (controls) participated in health examinations in the Medical Examination Center and 332 consecutive patients with the diagnosis of T2DM aged 18-80 years who were hospitalized in the Department of Endocrinology, Dalian Municipal Central Hospital Affiliated of Dalian Medical University, China were included from January to July 2018. Participants with incomplete data, histories of thyroid diseases, nondiabetic renal disease, infection, acute stress state, pregnancy, malignancies, and who were taking thyroid hormones and antithyroid agents were excluded. Among the 467 participants, 342 individuals met the inclusion criteria.

### 2.2. Clinical Examination and Laboratory Measurements

Information including family history, habits of smoking and drinking, duration of diabetes, medical histories of microvascular and macrovascular complications, and medication was obtained through the form of the standard questionnaire. Anthropometric measurements and physical examinations were performed by two trained endocrinologists. Body mass index (BMI) was calculated as weight (kg) divided by squared height (m). Blood pressure (BP) was detected on the right arm in a sitting position after a 10-minute rest period, and the mean of two successive systolic and diastolic BP (SBP/DBP) measurements was recorded. Hypertension was defined as SBP ≥ 140 mmHg and/or DBP ≥ 90 mmHg, or with positive histories of hypertension.

Blood samples were collected from participants at 5:00-6:00 am after 8 hours of overnight fast. Fasting plasma glucose (FPG), serum creatinine (SCr), blood urea nitrogen (BUN), serum total cholesterol (TC), serum triglyceride (TG), serum high-density lipoprotein cholesterol (HDL-C), and serum low-density lipoprotein cholesterol (LDL-C) were measured by routine laboratory methods (Siemens, ADVIA 2400, New York, US). Glycosylated hemoglobin (HbA1c) was determined by the high performance liquid chromatography method (TOSOH, HLC-723G8, Tokyo, Japan). Serum TSH, FT3, and FT4 were measured by chemiluminescence immunoassay (Siemens, ADVIA Centaur XP, New York, US). The reference ranges of TSH, FT3, and FT4 were 0.51-4.94 mIU/L, 3.5-6.5 pmol/L, and 11.5-22.7 pmol/L, respectively. Estimated glomerular filtration rate (eGFR) was calculated using the Modification of Diet in Renal Disease equation:

(1) eGFR  ml/min/1.73  m2=186×Scr88.4−1.154×age−0.203if  female,  ×0.742

Spot urinary samples of diabetic patients were collected at 7:00-8:00 am. Urinary albumin concentration was measured by nephelometry immunoassay (Beckmen Coulter, IMMAGE 800, California, US) and urinary creatinine concentration was measured by velocity method (Siemens, ADVIA 2400, New York, US). The average value of the urinary albumin-to-creatinine ratio (UACR) of three independent measurements was calculated. Microalbuminuria was defined as a UACR of 30-300 mg/g. Macroalbuminuria was defined as a UACR above 300 mg/g. DN was defined as the presence of microalbuminuria or macroalbuminuria.

### 2.3. Statistical Analysis

Continuous variables were described as mean ± SD for normally distributed data or median (inter quartile range) for nonnormally distributed data, and categorical variables were described as percentages. Comparisons of continuous data were performed via Student's *t*-test or one-way ANOVA for normally distributed variables, or Mann-Whitney rank sum test or Kruskal-Wallis one-way ANOVA for nonnormally distributed variables. And categorical data were compared via Pearson's Chi-square test. The correlations between TSH, FT3, and FT4 levels and related renal indexes were determined through Pearson correlation or Spearman correlation analysis. All tests were two-tailed and *p* value of < 0.05 was defined as statistically significant. Statistics were analyzed using SPSS 24.0 software.

## 3. Results

### 3.1. Clinical Characteristics of the Patients

Among 342 individuals recruited in this study, 103 (30.1%) were healthy volunteers, 100 (29.2%) were T2DM patients without DN, and 139 (40.6%) were diagnosed with DN. The comparisons of the clinical characteristics among controls and patients without and with DN are demonstrated in Table 1. No difference was found in gender or age among three groups (*p* = 0.601 and* p* = 0.211, respectively). Patients with DN experienced longer diabetic duration than those without DN (14.8±6.7 versus 7.5±5.3 years,* p *< 0.01). Both groups of patients without and with DN exhibited higher BMI, hip circumstance, HbA1c, and TG and lower HDL-C concentrations compared with controls (*p* < 0.01). Higher SCr, BUN, UACR, and TC levels and lower eGFR were found in patients with DN than those without DN (*p* < 0.05). Moreover, patients with DN showed significantly higher TSH and lower FT3 levels than those without DN (*p* < 0.01). FT4 concentrations demonstrated a descending trend among three groups while it did not reach statistical significance (*p* = 0.125).

### 3.2. Prevalence of SCH and Low T3 Syndrome in T2DM

As shown in Table 2, the prevalence of SCH was 10.8% in patients with DN, higher than the participants of controls (3.9%) and those without DN (2.0%) (*p* < 0.05). Meanwhile, patients with DN had a significantly higher prevalence of low T3 syndrome (20.9%) compared with participants in the control group (0%) and patients without DN (3.0%) (*p* < 0.01).

### 3.3. Correlations between Thyroid Function and Related Renal Indexes

As exhibited in Table 3, among patients without DN, a positive correlation was found in FT3 with SCr (*r* = 0.256,* p* = 0.010) and a negative correlation with UACR with statistical significance (*r* = -0.251,* p* = 0.012). No significant correlations were found between TSH or FT4 with these renal indexes.

Among patients with DN, there were positive correlations in TSH with SCr (*r* = 0.363,* p* = 0.013) and UACR (*r* = 0.337,* p* = 0.004), and FT3 as well as FT4 with eGFR with statistical significance (*r* = 0.560,* p* < 0.001 and* r* = 0.441,* p* < 0.001, respectively). TSH was negatively correlated with eGFR (*r* = -0.356,* p* < 0.001), while FT3 and FT4 were negatively correlated with SCr (*r* = -0.483,* p*< 0.001 and* r* = -0.407,* p* < 0.001, respectively) and UACR significantly (*r* = -0.522,* p*< 0.001 and* r* = -0.356,* p* < 0.001, respectively).

## 4. Discussion

Potential mechanisms of metabolic diseases have received increasing attention [20–23]. In this study, we included healthy individuals and patients of T2DM and exhibited evaluated level of TSH and decreased FT3 in T2DM patients with DN in a northeastern Chinese city for the first time to explore potential mechanism and management of DN. Also we estimated the prevalence of SCH and low T3 syndrome in the healthy group and T2DM patients without and with DN. We found that T2DM patients with DN are more likely to have SCH (prevalence of 10.8%) compared with healthy individuals (3.9%) and those without DN (2.0%), and the prevalence of low T3 syndrome demonstrated an increasing trend among groups of control (0%) and patients without DN (3%) and with DN (20.9%) with statistical significance.

There are complex interactions between thyroid function and kidney disease. Thyroid hormones are involved in the regulation of renal hemodynamics and glomerular filtration and influence the levels of SCr and glomerular filtration rate (GFR) via the effects on cardiac output, renal vascular function, and the renin-angiotensin-aldosterone system [24–28]. On the other hand, CKD affects thyroid hormonal synthesis through the hypothalamus-pituitary-thyroid axis and peripheral metabolism [29, 30]. According to previous studies, thyroid dysfunction was prevalent in CKD patients, including SCH and low T3 syndrome [31–33].

As DN has become the main cause of kidney failure and is significantly associated with increased morbidity and mortality [34, 35], the associations between thyroid dysfunction and T2DM as well as DN are drawing increasing concerns. A meta-analysis conducted by Han et al. [14] reported that the adjusted pooled prevalence of SCH in T2DM patients was 10.2% (95% CI 4.7–15.7%). Our finding indicated a prevalence rate of 7.1% of SCH in patients with T2DM, lower than the pooled prevalence of China (12.6%) and Europe (9.1%) in the above meta-analysis, but higher than that of Africa (4.7%) [14, 36, 37]. Reasons may be the differences in study design and characteristics of the participants, for instance, sex and age composition. Our study had a respectively higher proportion of male and younger patients compared with those included studies. In previous studies, it was observed that female and elder populations were more frequently suffered from SCH [14, 38]. Among DN patients, in a study on Japanese individuals the prevalence of SCH was 20.7% [39], which was higher than our result. Apart from the influence factors of study design, differences in race, environment, and life style may also explain the discrepancy. Meanwhile, the Japanese study also suggested an independent association between SCH and DN, and in the SCH group, eGFR was lower compared with the euthyroid group. Moreover, in the aforementioned Chinese report, subjects with SCH demonstrated significantly higher levels of UACR and SCr, and T2DM patients with SCH were associated with a high risk of DN (OR 3.15, 95% CI 1.48–6.69) [18]. These were in line with our result that subjects in DN group showed positive correlations in TSH with UACR (*r* = 0.337,* p* = 0.004) and SCr (*r* = 0.363,* p* = 0.013) and a negative correlation in TSH with eGFR (*r *= -0.356,* p* < 0.001). Conversely, in a study performed in Korea, investigators failed to find any associations between SCH and DN [40]. It should be noted that patients with thyroid disorders were not excluded, which might influence the results. In addition, the durations of diabetes in the Korean study were 6.9 ± 6.6 years in euthyroid patients and 8.9 ± 7.2 years in those who simultaneously had SCH and were shorter than those in our study (7.5 ± 5.3 years without DN and 14.8 ± 6.7 years with DN).

Furthermore, our study exhibited that levels of FT3 in T2DM (4.8 ± 0.7 pmol/L in patients without DN and 4.3 ± 0.9 pmol/L with DN) were lower than those of control group (5.2 ± 0.5 pmol/L), and the difference reached statistical significance (*p* < 0.01). In addition, the positive correlation in FT3 with eGFR and negative correlations with SCr and UACR implied the relationship between low FT3 levels and renal dysfunction. These data agree with the significant lower concentrations of FT3 in T2DM than the controls reported by Islam S et al. [41]. And according to analytic data from Wu et al. [16], subjects with DN had lower FT3 levels than those without DN with statistical significance (*p* < 0.01). Meanwhile, FT3 levels positively correlated with eGRF (*p* = 0.03) and negatively correlated with UACR (*p* < 0.01). After adjustment, results of multiple linear regression analysis implied an independent association between FT3 and UACR (*β* = −0.18, *t* = -3.70,* p* < 0.01).

The association between changes of serum thyroid hormones with DN may be explained by the following mechanisms. For SCH in DN, first, SCH is involved in the impairment of vascular function, which leads to renal vasoconstriction and peripheral circulation dysfunction. Functions of glomerular filtration and tubular thickening are damaged, and decreased GFR and the presence of urinary protein are observed [42, 43]. Second, in status of SCH the impairment of nitric oxide availability results in the damage of endothelial dilatation function, which is associated with the pathogenesis of DN [44, 45]. Third, thyroid hormone deficiencies in SCH lead to dyslipidemia and atherosclerosis, which indirectly harm renal function [46, 47]. For low T3 syndrome in DN, in diabetic patients there are changes in the regulation of hypothalamus-pituitary-thyroid axis. As central secretion of TRH and TSH is reduced and the conversion of T4 into T3 is suppressed in peripheral tissues, the levels of T3 decrease [48]. In addition, it was observed that the aforementioned functional alterations in vascular endothelium and dyslipidemia could be reversed through levothyroxine supplementation [49]. Meanwhile, as the majority of circulating thyroid hormones bound to protein, severe protein losses and hypoproteinemia in DN may lead to thyroid hormone depletion, commonly manifest low T3 levels [50].

There are several limitations in our study. First, as this is a cross-sectional study, we could not infer causal relationships between thyroid function and DN. Prospective studies are expected to confirm the associations between SCH as well as low T3 syndrome and DN in T2DM patients. Second, subjects included in this study were consecutive healthy volunteers and T2DM patients in a single hospital. Thus, the results may not be representative of the T2DM population in the northeast of China. Third, thyroid function, renal function, and blood fats tests were only measured once. For this reason, the results should not be overestimated in prevalence and the correlations.

Despite the limitations, this study suggested a potential relationship between thyroid dysfunction and DN in T2DM, thus implied the significance of routine monitoring of thyroid function, and provided a new view in the treatment of DN. American Association of Clinical Endocrinologists (AACE) and the American Thyroid Association (ATA) recommend regular monitoring of thyroid function in patients with type 1 diabetes mellitus (T1DM) [51]. According to the guidelines of European Thyroid Association, monitoring of thyroid function should be performed manually in T1DM [52]. However, currently relevant guidelines in patients with T2DM are lacking. Investigations are needed in the future to provide more evidence on the screening strategies of thyroid dysfunction in T2DM care. Furthermore, in the aspect of therapeutics, previous studies observed that levothyroxine therapy in DM improved thyroid function and renal indexes, while the mechanisms were indefinite [53, 54]. A recently published randomized and placebo-controlled study showed decreased levels of oxidative stress and injury of the kidney in early-staged DN patients with levothyroxine therapy [55]. However, the sample sizes were relatively small and the therapeutic durations were short in the above studies. Therefore, large-sampled interventional trials are expected in the future to explore the mechanisms and the effects of new therapies in DN patients.

## 5. Conclusion

Our study demonstrates high levels of TSH and low levels of FT3 in T2DM patients with DN. Besides, in DN patients TSH was positively correlated with SCr and UACR, and the correlations between FT3 with SCr and UACR were negative. The results may improve our understanding about the relationship between thyroid function and DN and thus may imply the necessity of regular monitoring of thyroid function in DN patients. Meanwhile, these results may provide evidence for new therapeutic strategies on DN in the future.

## Figures and Tables

**Table 1:** Comparisons of clinical characteristics between controls, patients without DN, and patients with DN.

	Controls (n = 103)	Patients without DN (n = 100)	Patients with DN (n = 139)	*p *value
Gender (male/female)	55/58	54/46	76/63	0.601
Age (years)	56.8±12.5	58.5±8.9	59.1±8.9	0.211
Diabetic duration (years)	-	7.5±5.3	14.8±6.7^d^	< 0.001
Hypertension (%)	41.7	47.0	70.5^bd^	0.001
Smoking history (%)	31.1	36.0	43.2	0.148
Drinking history (%)	43.7	48.0	51.8	0.458
BMI (kg/m^2^)	23.4±2.9	26.6±4.0^b^	26.1±4.1^b^	< 0.001
Waist circumstance (cm)	96.3±5.6	95. 8±9.9	98.9±10.0	0.079
Hip circumstance (cm)	85.0±8.6	101.1±9.9^b^	98.5±10.2^ad^	< 0.001
HbA1c (%)	5.3±0.5	8.1±2.1^b^	8.9±2.9^b^	< 0.001
SCr (*μ*mol/L)	67.5±17.6	57.0±12.2	228.0±113.9^ad^	< 0.001
eGFR (mL/min·1.73 m^2^)	103.0 (92.2-116.5)	123.5 (103.5-134.5)^b^	86.3 (14.6-117. 6)^bd^	< 0.001
BUN (mmol/L)	5.3±1.3	5.5±1.4	11.9±9.1^bd^	< 0.001
UACR (mg/g)	-	11.2 (6.9-16.0)	524.8 (92.8-3694.1)^d^	< 0.001
TC (mmol/L)	5.0±0.9	4.9±1.0	5.5±1.6^ac^	0.010
TG (mmol/L)	1.4 (0.9-1.9)	1.7 (1.2-2.5)^b^	1.8 (1.3-2.9)^b^	< 0.001
HDL-C (mmol/L)	1.4±0.4	1.1±0.2^b^	1.1±0.3^b^	< 0.001
LDL-C (mmol/L)	3.1±0.8	3.1±1.0	3.3±1.3	0.370
TSH (mIU/L)	1.9 (1.3-2.6)	2.0 (1.2-3.0)	2. 7 (1.8-3.7)^bd^	< 0.001
FT3 (pmol/L)	5.2±0.5	4.8±0.7^a^	4.3±0.9^bd^	< 0.001
FT4 (pmol/L)	15.4±1.8	15.2±2.1	14.9±2.2	0.125

DN, diabetic nephropathy; BMI, body mass index; HbA1c, glycosylated hemoglobin; SCr, serum creatinine; eGFR, estimated glomerular filtration rate; BUN, blood urea nitrogen; UACR, urinary albumin-to-creatinine ratio; TC, total cholesterol; TG, serum triglyceride; HDL-C, high-density lipoprotein cholesterol; LDL-C, low-density lipoprotein cholesterol. ^a^*p* < 0.05, ^b^*p* < 0.01 versus controls; ^c^*p* < 0.05, ^d^*p* < 0.01 versus patients without DN.

**Table 2:** Prevalence of SCH and low T3 syndrome.

	Controls (n = 103)	Patients without DN (n = 100)	Patients with DN (n = 139)	*P *Value
SCH	4 (3.9%)	2 (2.0%)	15(10.8%)^ac^	< 0.001
Low T3 Syndrome	0 (0%)	3 (3.0%)	29 (20.9%)^bd^	< 0.001

SCH, subclinical hypothyroidism. ^a^*p* < 0.05, ^b^*p* < 0.01 versus controls; ^c^*p* < 0.05, ^d^*p* < 0.01 versus patients without DN.

**Table 3:** Correlations between thyroid function and related renal indexes in T2DM patients without and with DN.

Parameters correlated	T2DM patients without DN		T2DM patients with DN	
	*r* value	*p* value	*r* value	*p* value
TSH with				
(i) SCr	-0.187	0.063	0.363	0.013
(ii) eGFR	-0.024	0.815	-0.356	< 0.001
(iii) UACR	0.017	0.863	0.337	0.004

FT3 with				
(i) SCr	0.256	0.010	-0.483	< 0.001
(ii) eGFR	0.034	0.736	0.560	< 0.001
(iii) UACR	-0.251	0.012	-0.522	< 0.001

FT4 with				
(i) SCr	0.155	0.125	-0.407	< 0.001
(ii) eGFR	0.020	0.846	0.441	< 0.001
(iii) UACR	0.012	0.909	-0.356	< 0.001

SCr, serum creatinine; eGFR, estimated glomerular filtration rate; UACR, urinary albumin-to-creatinine ratio.

## Data Availability

The data used to support the findings of this study are available from the corresponding author upon request.

## References

[1] Defronzo R. A. (2009). From the triumvirate to the ominous octet: a new paradigm for the treatment of type 2 diabetes mellitus. *Diabetes*.

[2] Wright E., Scism-Bacon J. L., Glass L. C. (2006). Oxidative stress in type 2 diabetes: the role of fasting and postprandial glycaemia. *International Journal of Clinical Practice*.

[3] Wild S., Roglic G., Green A., Sicree R., King H. (2004). Global prevalence of diabetes: estimates for the year 2000 and projections for 2030. *Diabetes Care*.

[4] Wang L., Gao P., Zhang M. (2017). Prevalence and Ethnic Pattern of Diabetes and Prediabetes in China in 2013. *Journal of the American Medical Association*.

[5] Zhang L., Long J., Jiang W. (2016). Trends in chronic kidney disease in China. *The New England Journal of Medicine*.

[6] Gregg E. W., Li Y., Wang J. (2014). Changes in diabetes-related complications in the United States, 1990–2010. *The New England Journal of Medicine*.

[7] Crunkhorn S., Patti M. E. (2008). Links between thyroid hormone action, oxidative metabolism, and diabetes risk?. *Thyroid*.

[8] Iwen K. A., Schröder E., Brabant G. (2013). Thyroid hormones and the metabolic syndrome. *European Thyroid Journal*.

[9] Han C., Xia X., Liu A. (2016). Circulating Betatrophin Is Increased in Patients with Overt and Subclinical Hypothyroidism. *Biomed Research Inteernational*.

[10] Han C., Rice M., Cai D. (2016). Neuroinflammatory and autonomic mechanisms in diabetes and hypertension. *American Journal of Physiology-Endocrinology and Metabolism*.

[11] Kalra S. (2014). Thyroid disorders and diabetes. *Journal of the Pakistan Medical Association*.

[12] Distiller L. A., Polakow E. S., Joffe B. I. (2014). Type 2 diabetes mellitus and hypothyroidism: The possible influence of metformin therapy. *Diabetic Medicine*.

[13] Villar H. C., Saconato H., Valente O. (2007). Thyroid hormone replacement for subclinical hypothyroidism. *Cochrane Database of Systematic Reviews*.

[14] Han C., He X., Xia X. (2015). Subclinical Hypothyroidism and Type 2 Diabetes: A Systematic Review and Meta-Analysis. *PLoS One*.

[15] Zhang Y., Chang Y., Ryu S. (2014). Thyroid hormone levels and incident chronic kidney disease in euthyroid individuals: the Kangbuk Samsung Health Study. *International Journal of Epidemiology*.

[16] Wu J., Li X., Tao Y. (2015). Free Triiodothyronine Levels Are Associated with Diabetic Nephropathy in Euthyroid Patients with Type 2 Diabetes. *International Journal of Endocrinology*.

[17] Chen L., Magliano D. J., Zimmet P. Z. (2012). The worldwide epidemiology of type 2 diabetes mellitus—present and future perspectives. *Nature Reviews Endocrinology*.

[18] Chen H.-S., Wu T.-E. J., Jap T.-S. (2007). Subclinical hypothyroidism is a risk factor for nephropathy and cardiovascular diseases in Type 2 diabetic patients. *Diabetic Medicine*.

[19] Zhao W., Han C., Shi X. (2014). Prevalence of goiter and thyroid nodules before and after implementation of the Universal Salt Iodization program in mainland China from 1985 to 2014: a systematic review and meta-analysis. *PLoS ONE*.

[20] Han C., Li C., Mao J. (2015). High Body Mass Index Is an Indicator of Maternal Hypothyroidism, Hypothyroxinemia, and Thyroid-Peroxidase Antibody Positivity during Early Pregnancy. *Biomed Research International*.

[21] Wang H., Lai Y., Han C. (2016). The Effects of Serum ANGPTL8/betatrophin on the Risk of Developing the Metabolic Syndrome – A Prospective Study. *Scientific Reports*.

[22] Han C., Wu W., Ale A., Kim M. S., Cai D. (2016). Central leptin and tumor necrosis factor-*α* (TNF*α*) in diurnal control of blood pressure and hypertension. *The Journal of Biological Chemistry*.

[23] Zhang Y., Reichel J. M., Han C., Zuniga-Hertz J. P., Cai D. (2017). Astrocytic Process Plasticity and IKK*β*/NF-*κ*B in Central Control of Blood Glucose, Blood Pressure, and Body Weight. *Cell Metabolism*.

[24] Karanikas G., Schütz M., Szabo M. (2004). Isotopic Renal Function Studies in Severe Hypothyroidism and after Thyroid Hormone Replacement Therapy. *American Journal of Nephrology*.

[25] Mariani L. H., Berns J. S. (2012). The renal manifestations of thyroid disease. *Journal of the American Society of Nephrology*.

[26] Kreisman S. H., Hennessey J. V. (1999). Consistent reversible elevations of serum creatinine levels in severe hypothyroidism. *JAMA Internal Medicine*.

[27] Ripoli A., Pingitore A., Favilli B. (2005). Does subclinical hypothyroidism affect cardiac pump performance? Evidence from a magnetic resonance imaging study. *Journal of the American College of Cardiology*.

[28] Villabona C., Sahun M., Roca M. (1999). Blood volumes and renal function in overt and subclinical primary hypothyroidism. *The American Journal of the Medical Sciences*.

[29] Kaptein E. M. (1996). Thyroid hormone metabolism and thyroid diseases in chronic renal failure. *Endocrine Reviews*.

[30] Singh P. A., Bobby Z., Selvaraj N., Vinayagamoorthi R. (2006). An evaluation of thyroid hormone status and oxidative stress in undialyzed chronic renal failure patients. *Indian Journal of Physiology and Pharmacology*.

[31] Song S. H., Kwak I. S., Lee D. W., Kang Y. H., Seong E. Y., Park J. S. (2009). The prevalence of low triiodothyronine according to the stage of chronic kidney disease in subjects with a normal thyroid-stimulating hormone. *Nephrology Dialysis Transplantation *.

[32] Lo J. C., Chertow G. M., Go A. S., Hsu C.-Y. (2005). Increased prevalence of subclinical and clinical hypothyroidism in persons with chronic kidney disease. *Kidney International*.

[33] Chonchol M., Lippi G., Salvagno G., Zoppini G., Muggeo M., Targher G. (2008). Prevalence of subclinical hypothyroidism in patients with chronic kidney disease. *Clinical Journal of the American Society of Nephrology*.

[34] Tung C., Hsu Y., Shih Y., Chang P., Lin C. (2018). Glomerular mesangial cell and podocyte injuries in diabetic nephropathy. *Nephrology*.

[35] Fouli G., Gnudi L. (2018). The Future: Experimental Therapies for Renal Disease in Diabetes. *Nephron*.

[36] Diez J. J., Sanchez P., Iglesias P. (2011). Prevalence of thyroid dysfunction in patients with type 2 diabetes. *Experimental and Clinical Endocrinology Diabetes*.

[37] Ghazali S. M., Abbiyesuku F. M. (2010). Thyroid dysfunction in type 2 diabetics seen at the university college hospital, ibadan, nigeria. *Nigerian Journal of Physiological Sciences*.

[38] Hollowell J. G., Staehling N. W., Dana Flanders W. (2002). Serum TSH, T(4), and thyroid antibodies in the United States population (1988 to 1994): National Health and Nutrition Examination Survey (NHANES III). *The Journal of Clinical Endocrinology & Metabolism*.

[39] Furukawa S., Yamamoto S., Todo Y. (2014). Association between subclinical hypothyroidism and diabetic nephropathy in patients with type 2 diabetes mellitus. *Endocrine Journal*.

[40] Yang J.-K., Liu W., Shi J., Li Y.-B. (2010). An association between subclinical hypothyroidism and sight-threatening diabetic retinopathy in type 2 diabetic patients. *Diabetes Care*.

[41] Islam S., Yesmine S., Khan S. A., Alam N. H., Islam S. (2008). A comparative study of thyroid hormone levels in diabetic and non-diabetic patients. *Southeast Asian Journal of Tropical Medicine and Public Health*.

[42] Asmah B. J., Wan Nazaimoon W. M., Norazmi K., Tan T. T., Khalid B. A. K. (1997). Plasma Renin and Aldosterone in Thyroid Diseases. *Hormone and Metabolic Research*.

[43] Bradley S. E., Coelho J. B., Sealey J. E., Edwards K. D. G., Stéphan F. (1982). Changes in glomerulotubular dimensions, single nephron glomerular filtration rates and the renin-angiotensin system in hypothyroid rats. *Life Sciences*.

[44] Schalkwijk C. G., Stehouwer C. D. A. (2005). Vascular complications in diabetes mellitus: the role of endothelial dysfunction. *Clinical Science*.

[45] Dinneen S. F., Gerstein H. C. (1997). The association of microalbuminuria and mortality in non-insulin-dependent diabetes mellitus: a systematic overview of the literature. *JAMA Internal Medicine*.

[46] Cappola A. R., Ladenson P. W. (2003). Hypothyroidism and atherosclerosis. *The Journal of Clinical Endocrinology & Metabolism*.

[47] Hak A. E., Pols H. A. P., Visser T. J., Drexhage H. A., Hofman A., Witteman J. C. M. (2000). Subclinical hypothyroidism is an independent risk factor for atherosclerosis and myocardial infarction in elderly women: the Rotterdam study. *Annals of Internal Medicine*.

[48] Udiong C. E. J., Udoh A. E., Etukudoh M. E. (2007). Evaluation of thyroid function in diabetes mellitus in Calabar, Nigeria. *Indian Journal of Clinical Biochemistry*.

[49] Taddei S., Caraccio N., Virdis A. (2003). Impaired endothelium-dependent vasodilatation in subclinical hypothyroidism: beneficial effect of levothyroxine therapy. *The Journal of Clinical Endocrinology & Metabolism*.

[50] Feinstein E. I., Kaptein E. M., Nicoloff J. T., Massry S. G. (1982). Thyroid function in patients with nephrotic syndrome and normal renal function. *American Journal of Nephrology*.

[51] Garber J. R., Cobin R. H., Gharib H. (2012). Erratum: Clinical practice guidelines for hypothyroidism in adults: Cosponsored by the American association of clinical endocrinologists and the American thyroid association. *Thyroid*.

[52] Pearce S. H., Brabant G., Duntas L. H. (2013). 2013 ETA guideline: management of subclinical hypothyroidism. *European Thyroid Journal*.

[53] Adrees M., Gibney J., El-Saeity N., Boran G. (2009). Effects of 18 months of l-T4 replacement in women with subclinical hypothyroidism. *Clinical Endocrinology*.

[54] Shin D. H., Lee M. J., Lee H. S. (2013). Thyroid hormone replacement therapy attenuates the decline of renal function in chronic kidney disease patients with subclinical hypothyroidism. *Thyroid*.

[55] Chen Y., Wu G., Xu M. (2018). The effect of l-thyroxine substitution on oxidative stress in early-stage diabetic nephropathy patients with subclinical hypothyroidism: a randomized double-blind and placebo-controlled study. *International Urology and Nephrology*.

